# Correction: Assessment of the toxicity and inflammatory effects of different-sized zinc oxide nanoparticles in 2D and 3D cell cultures

**DOI:** 10.1039/d0ra90118k

**Published:** 2020-12-16

**Authors:** Zhipan Wu, Rongfa Guan, Miao Tao, Fei Lyu, Guozhou Cao, Mingqi Liu, Jianguo Gao

**Affiliations:** Zhejiang Provincial Key Laboratory of Biometrology and Inspection & Quarantine, College of Life Sciences, China Jiliang University Xueyuan Road 258 Hangzhou 310018 China wuzhipanjl@126.com taomfood@126.com mqliu524@163.com Rongfaguan@163.com +86-571-8691449; Ocean College, Zhejiang University of Technology Hangzhou 310014 China lvfei_zju@163.com; Ningbo Entry–Exit Inspection and Quarantine Technology Center Ningbo 315000 China caogz@nbciq.gov.cn; Inspection and Quarantine Center of Shandong Exit & Entry Inspection and Quarantine Bureau Qingdao 266002 Shandong Province China china.gjg@163.com

## Abstract

Correction for ‘Assessment of the toxicity and inflammatory effects of different-sized zinc oxide nanoparticles in 2D and 3D cell cultures’ by Zhipan Wu, Rongfa Guan, Miao Tao *et al.*, *RSC Adv.*, 2017, **7**, 12437–12445, DOI: 10.1039/C6RA27334C.

The authors regret that in Fig. 1a, while the correct material is shown, the incorrect representative image was chosen, in which the overall distribution of nanoparticles was not clear. The correct image is given here.

**Fig. 1:**
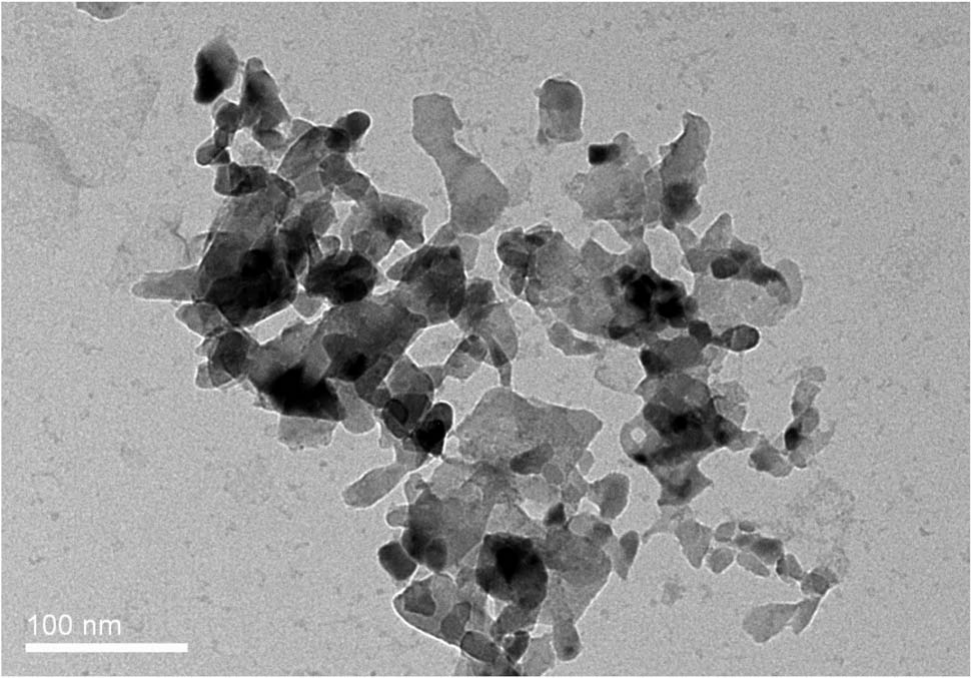
(A) Microscopy characterizations of ZnO NPs. TEM images of an average: (a) 24 nm ZnO NPs (the average particle size).

The Royal Society of Chemistry apologises for these errors and any consequent inconvenience to authors and readers.

## Supplementary Material

